# Cerebral Amyloid Angiopathy Presenting as Massive Subarachnoid Haemorrhage: A Case Study and Review of Literature

**DOI:** 10.3389/fnagi.2020.538456

**Published:** 2020-11-10

**Authors:** Satoshi Saito, Yoshihiko Ikeda, Daisuke Ando, Roxana Octavia Carare, Hatsue Ishibashi-Ueda, Masafumi Ihara

**Affiliations:** ^1^Clinical Neurosciences, Faculty of Medicine, University of Southampton, Southampton, United Kingdom; ^2^Department of Neurology, National Cerebral and Cardiovascular Center, Suita, Japan; ^3^Department of Pediatric Dentistry, Graduate School of Dentistry, Osaka University, Suita, Japan; ^4^Research Fellow of Japan Society for the Promotion of Science, Tokyo, Japan; ^5^Department of Pathology, National Cerebral and Cardiovascular Center, Suita, Japan

**Keywords:** case report, intracerebral haemorrhage, subarachnoid haemorrhage, cerebral amyloid angiopathy, pathology

## Abstract

Cerebral amyloid angiopathy (CAA) is characterised by the progressive accumulation of β-amyloid (Aβ) in the walls of cerebral capillaries and arteries representing a major cause of haemorrhagic stroke including lobar intracerebral haemorrhage (ICH) and convexity subarachnoid haemorrhage (SAH). Haemorrhaging from CAA predominantly involves smaller arteries rather than arterial aneurysm. Restricted bleeding into the subarachnoid space in CAA results in asymptomatic or mild symptomatic SAH. Herein, we present an autopsied case of massive SAH related to CAA. An 89-year-old male with a history of mild Alzheimer’s disease (AD) and advanced pancreatic cancer with liver metastasis developed sudden onset of coma. Head CT illustrated ICH located in the right frontal lobe and right insula, as well as SAH bilaterally spreading from the basal cistern to the Sylvian fissure, with hydrocephalus and brain herniation. He died about 24 h after onset and the post-mortem examination showed no evidence of arterial aneurysm. The substantial accumulation of Aβ in the vessels around the haemorrhagic lesions led to the diagnosis of ICH related to CAA and secondary SAH, which may have been aggravated by old age and malignancy. This case suggests that CAA can cause severe SAH resembling aneurysmal origin and thus may be overlooked when complicated by atypical cerebral haemorrhage.

## Introduction

Cerebral amyloid angiopathy (CAA) is a cerebrovascular amyloidosis and a known cause of haemorrhagic stroke. Seven amyloid proteins have so far been reported in CAA including β-amyloid (Aβ), cystatin C, transthyretin, gelsolin, prion protein, ABri/ADan, and immunoglobulin light-chain amyloid (Yamada, 2015). The most common form is Aβ-type CAA, which is frequently concomitant with Alzheimer’s disease (AD; Love et al., 2014).

Cerebrovascular Aβ accumulation induces smooth muscle cell degeneration and vessel wall thickening, resulting in variable degrees of intracerebral haemorrhage (ICH; Love et al., 2014). Bleeding into the subarachnoid space is also common in CAA, presenting as convexity subarachnoid haemorrhage (SAH) in acute and superficial siderosis in the chronic phase. However, CAA is seldom described as a cause of massive SAH resembling aneurysmal rupture (Ohshima et al., 1990; Charidimou et al., 2015; Ni et al., 2015; Raposo et al., 2018). Here, we report an autopsied case of widespread SAH related to CAA.

## Case Description

**Figure 1:**
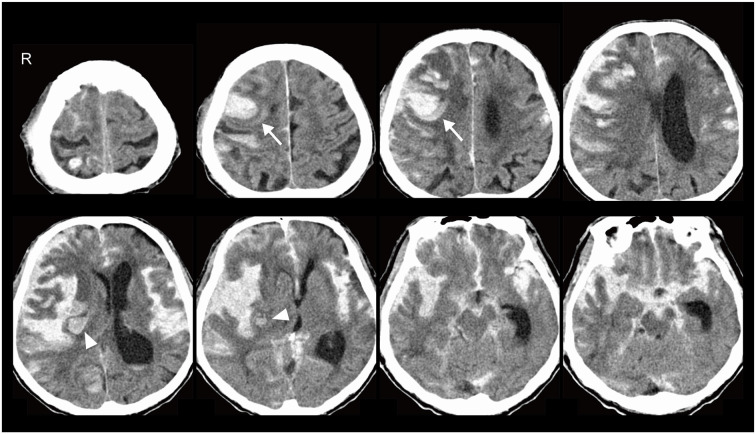
Head CT showed right frontal lobe (arrows) and insula (arrowheads) intracerebral haemorrhage (ICH) adjacent to the brain surface. Blood was also noted in the subarachnoid space, spreading from the basal cistern to the bilateral Sylvian fissure with hydrocephalus and brain herniation.

An 89-year-old male was admitted to our hospital due to sudden onset of coma. Pancreatic cancer with liver metastasis was diagnosed 8 months before admission, and palliative care had been performed. He had a history of mild AD at the age of 88 and brainstem haemorrhage at 82. He was able to look after his own affairs without assistance just before the admission. Amlodipine besilate, sitagliptin phosphate hydrate and febuxostat were administered for hypertension, diabetes mellitus and hyperuricemia. Heavy drinking and smoking in middle age was reported by the family of the patient. Blood pressure and pulse rate were 135/78 mmHg and 92/min. Glasgow Coma Scale score was 6/15 (E4V1M1). Conjugate gaze deviation to the right, anisocoria, facial nerve palsy on the left side and urinary retention were noted. National Institutes of Health Stroke Scale was 37/42. Head CT showed right frontal lobe and insula ICH adjacent to the brain surface (Figure 1). Haematoma volume was estimated as 14.9 and 5.9 cm^3^, respectively (manual segmentation using OsiriX software: Pixmeo, Bernex, Switzerland). Blood was also noted in the subarachnoid space, spreading from the basal cistern to the bilateral Sylvian fissure with hydrocephalus and brain herniation. MR/CT angiography and digital subtraction angiogram were not performed based on the living will. Complete blood count showed decreased haemoglobin (11.0 g/dl) and haematocrit concentration (33.4%), elevated white blood cells (14,100/μl) and normal level of platelets (183,000/μl). Coagulation assays were normal except for elevated d-dimer (193.6 μl/ml). Slightly decreased renal function (estimated glomerular filtration rate: 67.4 ml/min/1.73 m^2^) and elevated level of blood glucose (281 mg/dl) were observed. Hepatic function was normal. He was diagnosed as ICH with secondary SAH and subsequently died approximately 24 h after onset.

**Figure 2:**
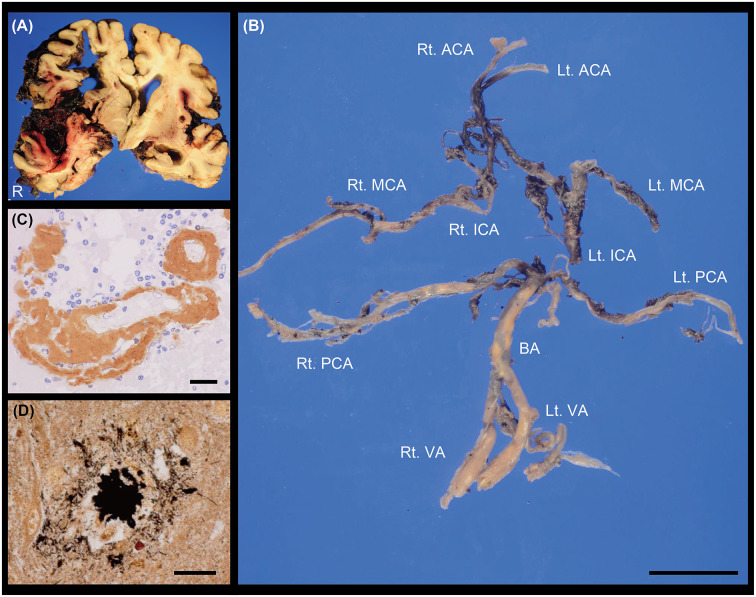
**(A)** Coronal section of the brain. ICH with subarachnoid haemorrhage (SAH) extension was observed in the right hemisphere. **(B)** Macroscopic image showing the cerebral vasculature. We did not find any evidence of saccular aneurysm in these vessels. The posterior communicating arteries were not identified. **(C)** Cerebrovascular immunostaining of Aβ (brown; M0872, DAKO, 1:50) with nuclear counterstain (blue). **(D)** Senile plaque with modified Bielschowsky staining. Scale bars indicate 2 cm **(B)** and 20 μm **(C,D)**. ACA, anterior cerebral artery; BA, basilar artery; ICA, internal carotid artery; Lt, left; MCA, middle cerebral artery; PCA, posterior cerebral artery; Rt, right; VA, vertebral artery.

At autopsy, the total brain weight, including intracranial haematoma, was 1,285 g. Acute ICH was observed in the right frontal lobe and insula. Bleeding into the subarachnoid space widely spread to the sulcus of the frontotemporal cortex in the right hemisphere, accompanied by an uncal and subfalcine hernia (Figure 2A). We did not find any aneurysm in the cerebral vasculature (Figure 2B). Histologically, abundant Aβ deposits were observed within the leptomeningeal and cortical arteries around the parenchymal haemorrhagic lesions (Figure 2C), which led to the pathological diagnoses of ICH related to CAA with SAH extension. Periarterial spaces were enlarged around the ICH. There were no other lesions contributing to the bleeding, including brain metastasis. Modified Bielschowsky staining uncovered senile plaques and neurofibrillary tangles (Figure 2D), which was compatible with the diagnosis of AD (Hyman et al., 2012); Aβ plaque score, A1 (Thal et al., 2002), neurofibrillary tangle stage, B2 (Braak and Braak, 1991), neuritic plaque score, C3 (Mirra et al., 1991).

Written informed consent for autopsy and the publication was obtained from his legal representatives.

## Discussion

The presented case was CAA-related ICH accompanied by massive SAH, although the volume of parenchymal haematoma was relatively small (Patel et al., 2009). Severe SAH resembling that of aneurysmal origin is rare in CAA (Ohshima et al., 1990; Charidimou et al., 2015; Ni et al., 2015; Raposo et al., 2018). Old age and pancreatic cancer may have exacerbated the bleeding from the Aβ-positive vessels into the subarachnoid space.

SAH is a life-threatening cerebrovascular disease with a high mortality rate. It accounts for only 3% of all strokes, but for 5% of stroke deaths and more than one-quarter of potential life years lost through stroke (van Gijn and Rinkel, 2001). More than 80% of SAH arise from the rupture of saccular aneurysms. The findings of digital subtraction angiogram are well correlated with the pathology (Smith, 1963; Smith et al., 1983). The angiogram is therefore regarded as the gold standard for aneurysm detection. Approximately 30% of aneurysmal SAH induce ICH (van Gijn and Rinkel, 2001). SAH is also caused by a variety of conditions including perimesencephalic haemorrhage, arterial dissection, cerebral arteriovenous malformation, dural arteriovenous fistula, vascular lesions around the spinal cord, septic aneurysm, pituitary apoplexy, cocaine abuse and trauma (van Gijn and Rinkel, 2001). CAA occasionally induce convexity SAH, which are usually asymptomatic or mild symptomatic, although the risk of future intracranial haemorrhage and death of patients with CAA-convexity SAH is very high (Calviere et al., 2019). Convexity SAH is excluded if the involvement of the adjoining brain parenchyma is observed (Kumar et al., 2010).

Approximately 40% of ICH cases are associated with moderate or severe CAA in the UK (Rodrigues et al., 2018). Lobar, but not deep, ICH is especially related to CAA (Rodrigues et al., 2018). The extension of SAH is a frequent finding and recognized in about 80% of CAA-ICH cases (Rodrigues et al., 2018; Renard et al., 2019), which could be attributed to CAA predominantly affecting leptomeningeal and cortical arteries compared to intracortical arteries and capillaries (Takeda et al., 2003; Thal et al., 2008). Both the Boston MRI and Edinburgh CT-based diagnostic criteria are now available for the diagnosis of CAA-ICH (Greenberg and Charidimou, 2018; Rodrigues et al., 2018). However, CAA is likely to be clinically underdiagnosed due to multiple clinical phenotypes, especially in the elderly (Sakai et al., 2019; Fakan et al., 2020). Early diagnosis of CAA is important for guiding prognosis and treatment decisions. A recent prospective study (a median follow-up time of 2.5 years) showed progression to dementia was found in more than a quarter of patients with CAA-ICH, even if no dementia was recognized after the acute phase of ICH (Xiong et al., 2019). High ICH recurrence rate was also reported in patients with CAA-ICH, compared to other forms of ICH (Pasi et al., 2018).

In conclusion, this case demonstrates that CAA-ICH can mimic severe SAH resembling that of aneurysmal origin, suggesting that CAA induces a wider spectrum of cerebrovascular disorders than previously expected.

## Data Availability Statement

All datasets generated for this study are included in the article.

## Ethics Statement

Written informed consent for autopsy and the publication of this case report was obtained from his legal representatives.

## Author Contributions

SS and MI contributed to the conceptualisation and writing of the first draft. YI and HI-U performed pathological evaluation. YI, DA, RC, and HI-U critically reviewed and edited this manuscript. All authors contributed to the article and approved the submitted version.

## Conflict of Interest

The authors declare that the research was conducted in the absence of any commercial or financial relationships that could be construed as a potential conflict of interest.

## References

[B1] Braak H. Braak E. (1991). Neuropathological stageing of Alzheimer-related changes. Acta Neuropathol. 82, 239–259. 10.1007/bf00308809 1759558

[B2] Calviere L. Viguier A. Patsoura S. Rousseau V. Albucher J. F. Planton M.. (2019). Risk of intracerebral hemorrhage and mortality after convexity subarachnoid hemorrhage in cerebral amyloid angiopathy. Stroke 50, 2562–2564. 10.1161/STROKEAHA.119.026244 31337297

[B3] Charidimou A. Linn J. Vernooij M. W. Opherk C. Akoudad S. Baron J. C.. (2015). Cortical superficial siderosis: detection and clinical significance in cerebral amyloid angiopathy and related conditions. Brain 138, 2126–2139. 10.1093/brain/awv162 26115675

[B4] Fakan B. Reisz Z. Zadori D. Vecsei L. Klivenyi P. Szalardy L. (2020). Predictors of localization, outcome and etiology of spontaneous intracerebral hemorrhages: focus on cerebral amyloid angiopathy. J. Neural Transm. 127, 963–972. 10.1007/s00702-020-02174-2 32193732 PMC7248013

[B5] Greenberg S. M. Charidimou A. (2018). Diagnosis of cerebral amyloid angiopathy: evolution of the boston criteria. Stroke 49, 491–497. 10.1161/STROKEAHA.117.016990 29335334 PMC5892842

[B6] Hyman B. T. Phelps C. H. Beach T. G. Bigio E. H. Cairns N. J. Carrillo M. C.. (2012). National institute on aging-Alzheimer’s association guidelines for the neuropathologic assessment of Alzheimer’s disease. Alzheimers Dement. 8, 1–13. 10.1016/j.jalz.2011.10.007 22265587 PMC3266529

[B7] Kumar S. Goddeau R. P. Jr. Selim M. H. Thomas A. Schlaug G. Alhazzani A.. (2010). A traumatic convexal subarachnoid hemorrhage: clinical presentation, imaging patterns and etiologies. Neurology 74, 893–899. 10.1212/WNL.0b013e3181d55efa 20231664 PMC2836868

[B8] Love S. Chalmers K. Ince P. Esiri M. Attems J. Jellinger K.. (2014). Development, appraisal, validation and implementation of a consensus protocol for the assessment of cerebral amyloid angiopathy in post-mortem brain tissue. Am. J. Neurodegener. Dis. 3, 19–32. 24754000 PMC3986608

[B9] Mirra S. S. Heyman A. McKeel D. Sumi S. M. Crain B. J. Brownlee L. M. (1991). The consortium to establish a registry for Alzheimer’s disease (CERAD). Part II: Standardization of the neuropathologic assessment of Alzheimer’s disease. Neurology 41, 479–486. 10.1212/wnl.41.4.479 2011243

[B10] Ni J. Auriel E. Jindal J. Ayres A. Schwab K. M. Martinez-Ramirez S.. (2015). The characteristics of superficial siderosis and convexity subarachnoid hemorrhage and clinical relevance in suspected cerebral amyloid angiopathy. Cerebrovasc. Dis. 39, 278–286. 10.1159/000381223 25871492 PMC4458203

[B11] Ohshima T. Endo T. Nukui H. Ikeda S. Allsop D. Onaya T. (1990). Cerebral amyloid angiopathy as a cause of subarachnoid hemorrhage. Stroke 21, 480–483. 10.1161/01.str.21.3.480 2309274

[B12] Pasi M. Charidimou A. Boulouis G. Auriel E. Ayres A. Schwab K. M.. (2018). Mixed-location cerebral hemorrhage/microbleeds: underlying microangiopathy and recurrence risk. Neurology 90, e119–e126. 10.1212/WNL.0000000000004797 29247070 PMC5772153

[B13] Patel P. V. FitzMaurice E. Nandigam R. N. Auluck P. Viswanathan A. Goldstein J. N.. (2009). Association of subdural hematoma with increased mortality in lobar intracerebral hemorrhage. Arch. Neurol. 66, 79–84. 10.1001/archneur.66.1.79 19139303 PMC3085991

[B14] Raposo N. Calviere L. Cazzola V. Planton M. Patsoura S. Wargny M.. (2018). Cortical superficial siderosis and acute convexity subarachnoid hemorrhage in cerebral amyloid angiopathy. Eur. J. Neurol. 25, 253–259. 10.1111/ene.13484 29053885

[B15] Renard D. Parvu T. Thouvenot E. (2019). Finger-like projections in lobar haemorrhage on early magnetic resonance imaging is associated with probable cerebral amyloid angiopathy. Cerebrovasc. Dis. 47, 121–126. 10.1159/000499032 31063997

[B16] Rodrigues M. A. Samarasekera N. Lerpiniere C. Humphreys C. McCarron M. O. White P. M.. (2018). The edinburgh CT and genetic diagnostic criteria for lobar intracerebral haemorrhage associated with cerebral amyloid angiopathy: model development and diagnostic test accuracy study. Lancet Neurol. 17, 232–240. 10.1016/S1474-4422(18)30006-1 29331631 PMC5818029

[B17] Sakai K. Ueda M. Fukushima W. Tamaoka A. Shoji M. Ando Y. (2019). Nationwide survey on cerebral amyloid angiopathy in Japan. Eur. J. Neurol. 26, 1487–1493. 10.1111/ene.14031 31232495

[B18] Smith B. (1963). Cerebral pathology in subarachnoid haemorrhage. J. Neurol. Neurosurg. Psychiatry 26, 535–539. 10.1136/jnnp.26.6.535 14083227 PMC495633

[B19] Smith R. R. Clower B. R. Peeler D. F. Jr. Yoshioka J. (1983). The angiopathy of subarachnoid hemorrhage: angiographic and morphologic correlates. Stroke 14, 240–245. 10.1161/01.str.14.2.240 6836650

[B20] Takeda S. Yamazaki K. Miyakawa T. Onda K. Hinokuma K. Ikuta F.. (2003). Subcortical hematoma caused by cerebral amyloid angiopathy: Does the first evidence of hemorrhage occur in the subarachnoid space? Neuropathology 23, 254–261. 10.1046/j.1440-1789.2003.00506.x 14719539

[B21] Thal D. R. Griffin W. S. de Vos R. A. Ghebremedhin E. (2008). Cerebral amyloid angiopathy and its relationship to Alzheimer’s disease. Acta Neuropathol. 115, 599–609. 10.1007/s00401-008-0366-2 18369648

[B22] Thal D. R. Rub U. Orantes M. Braak H. (2002). Phases of Aβ-deposition in the human brain and its relevance for the development of AD. Neurology 58, 1791–1800. 10.1212/wnl.58.12.1791 12084879

[B23] van Gijn J. Rinkel G. J. (2001). Subarachnoid haemorrhage: diagnosis, causes and management. Brain 124, 249–278. 10.1093/brain/124.2.249 11157554

[B24] Xiong L. Charidimou A. Pasi M. Boulouis G. Pongpitakmetha T. Schirmer M. D.. (2019). Predictors for late post-intracerebral hemorrhage dementia in patients with probable cerebral amyloid angiopathy. J. Alzheimers Dis. 71, 435–442. 10.3233/JAD-190346 31403947 PMC9301963

[B25] Yamada M. (2015). Cerebral amyloid angiopathy: emerging concepts. J. Stroke 17, 17–30. 10.5853/jos.2015.17.1.17 25692104 PMC4325636

